# The Allocation of Water Resources in the Midstream of Heihe River for the “97 Water Diversion Scheme” and the “Three Red Lines”

**DOI:** 10.3390/ijerph18041887

**Published:** 2021-02-16

**Authors:** Wenjie Geng, Xiaohui Jiang, Yuxin Lei, Jinyan Zhang, Huan Zhao

**Affiliations:** 1 Shaanxi Key Laboratory of Earth Surface System and Environmental Carrying Capacity, College of Urban and Environmental Sciences, Northwest University, Xi’an 710127, China; gengwenjie@stumail.nwu.edu.cn (W.G.); leiyuxin@stumail.nwu.edu.cn (Y.L.); zhangjinyan@stumail.nwu.edu.cn (J.Z.); zhaohuan@stumail.nwu.edu.cn (H.Z.); 2 Department of Physical Geography, College of Urban and Environmental Science, Northwest University, Xi’an 710127, China

**Keywords:** The “97 Water Diversion Scheme”, The “Three Red Lines”, ecological water, water resources allocation model, Heihe River

## Abstract

Rapid economic and societal development increases resource consumption. Understanding how to balance the discrepancy between economic and social water use and ecological water use is an urgent problem to be solved, especially in arid areas. The Heihe River is the second-largest inland river in China, and this problem is notable. To ensure the downstream ecological water use, the “Water Distribution Plan for the Mainstream of the Heihe River” (97 Water Diversion Scheme) controls the discharge of Yingluo Gorge and Zhengyi Gorge, while the “Opinions of applying the strictest water resources control system” (Three Red Lines) restricts the water use. With the development of the economy and agriculture in the midstream, Zhengyi Gorge’s discharge cannot reach the Heihe River’s ecological water downstream. This paper is under the constraints of the “97 Water Diversion Scheme” of Heihe River and the “Three Red Lines” of the total water use control index for Zhangye County. We constructed a water resource allocation model for the midstream of Heihe River to reasonably allocate water resources in the Heihe River’s midstream and downstream. This model is divided into three parts: Establish the mathematical equation, simulate the water consumption under the different inflow conditions, and ensure each water user’s demand. The result showed that if we fail to confine total water consumption in the midstream, through the reasonable allocation of water resources, the real water use and water consumption of the middle Heihe River will be greater than the “97 Water Diversion Scheme” and the “Three Red Lines.” If we confine water consumption, they will be within the “97 Water Diversion Scheme” and the “Three Red Lines,” at the same time, they can reach the downstream of the Heihe River’s ecological water. Besides, under the premise of satisfying the economic water and ecological water downstream of the Heihe River, returning farmland to wasteland and strengthening water-saving measures will improve water efficiency and be more conducive to allocating water resources.

## 1. Introduction

Water resources are essential to all life, primary natural resources, strategic economic resources, and ecological control factors [1,2,3]. Moreover, many countries face inevitable conflicts among the financial and the environment caused by limited water resources, especially in arid and semi-arid areas [4,5,6,7,8,9,10]. Arid regions (including semi-arid areas) account for one-third of the global land area, are spread over more than 50 countries and regions, and account for 15% of the world’s population. Thus, water competition in arid and semi-arid basins is becoming more intense [11,12], for example, in the Aral Sea Basin [13], the Tarim River Basin [14], the Colorado River basin [10], and the Heihe River Basin [15]. Attempts to address natural resource development without considering many other development issues within watersheds will often fail [16,17,18]. Besides, the waste of water resources has exacerbated the water shortage crisis [19,20]. About 90% of freshwater resources in northwest China are used for agricultural irrigation, characterized by low precipitation and high evaporation. In contrast, the average utilization coefficient of irrigation water is about 0.5 [21,22,23,24]. This utilization of agricultural water resources is insufficient, and the water supply cannot fully meet the demand. Thus the gap between water supply and demand has become a significant impediment to economic development, especially for areas facing severe water shortages [6,21,25]. Water allocation is one of the most effective water management tools for dealing with this conflict. Hence, the water resources’ optimal allocation problem has been of interest to water resource managers and researchers [3,22,25,26,27,28,29,30,31,32,33,34,35]. By building a model, stakeholders can express their points of view, learn about other perspectives, and examine factual knowledge and subjective perceptions [36].

The Heihe River is the second-largest inland river in China. It is an essential water source in Northwest China; it is also the study area’s primary water source. The runoff from Yingluo Gorge is the main water source of the midstream of the Heihe River. The discharge from Zhengyi Gorge determines the ecological water demand downstream of the Heihe River. If the discharge cannot reach the Heihe River’s ecological water consumption downstream, it will affect the Heihe River’s ecological environment. With the rapid development of the economy and agriculture in the midstream, water resources demand has increased significantly [37,38,39]. This increase in water consumption in the middle reaches impacted the downstream’s ecology [15,19,40]. In 1992, to solve the water allocation problem between the financial and the environment, and the water conflict between the midstream and downstream [41,42], the former State Planning Commission approved a water distribution scheme for the Heihe River mainstream under the average condition of many years, that is, the “92 Water Distribution Scheme”. However, as a result of the “92 Water Distribution Scheme”, the discharge volume of Zhengyi Gorge cannot meet the ecological water demand downstream, which leads to the deterioration of the ecological environment downstream of Heihe River. In 1997, with the State Council’s approval, the Ministry of Water Resources approved the “Heihe River Main Stream Water Allocation Plan”—the “97 Water Diversion Scheme.” Since implementing the “Heihe River Main Stream Water Allocation Plan,” Yingluo Gorge and Zhengyi Gorge’s discharge has been strictly controlled, thus alleviating the downstream’s ecological water shortage.

To effectively curb the excessive development and utilization of water resources, China’s government established the “Three Red Lines” system that includes total water use control, water efficiency, and water functional areas that limit pollution capacity [43,44,45]. The General Office of the People’s Government of Gansu Province issues the “Notice on the Issuance of Water Resource Management and Control Targets of Gansu Province’s prefecture-level Administrative Regions in 2020 and 2030”, which specifies targets for water resource management and control in Zhangye. According to the Heihe River water diversion plan, the county (district) water resources allocation plan, and actual water use, the Zhangye Municipal People’s government promulgated the total water use index Zhangye county-level administrative region in 2015, 2020, and 2030. By controlling the whole district and the county’s water consumption, we can achieve the water’s overall control.

With the continued development of the economy and increasing demand for water resources, problems in controlling total water use and the “97 Water Diversion Scheme” gradually appeared [44,46]. In recent years, improvements in the water-saving level in the midstream and the irrigated area increase. When the total water use changes only slightly, water consumption will increase to a certain extent, and this may result in real water use not exceeding the “Three Red Lines”, but still exceeding the level set by the “97 Water Diversion Scheme”, caused ecological water reduction in the downstream of Heihe River [37,47,48,49]. To address this discrepancy, many solutions have been proposed; Ge built a model to allocating water based on water requirements and equity to help multi-level decision makers manage water resources in a Decision Support System (DSS) while fully accounting for the effects of human activities [50]; Zhang simulated the change in economic losses under the economic priority (EP) scenario via computable general equilibrium (CGE) modeling and that of the ecological area under the eco-environmental sustainability (ES) scenario by ecological water demand modeling [51]; Li studied the optimal MCRC modes for irrigation systems, consisting of both agricultural irrigation and eco-logical irrigation, based on the optimal ways, an inexact multi-stage programming stochastic programming (IMSP) model under uncertainty will be developed for irrigation water allocation considering ecological environment protection [52]; Pan coupled the canal water distribution optimization model and soil moisture simulation model to build a two-level canal water distribution optimization model based on canal water transfer simulation and soil water balance simulation [53]; Wang constructed a multi-objective optimization model of water resources under certain conditions and based on Me measure constraints [54]. However, they don’t consider the “97 Water Diversion Scheme” and “Three Red Lines” simultaneously, allocating water consumption in the midstream to meet the downstream ecological water consumption.

In this paper, we constructed a water resource allocation model for Heihe River based on the “97 Water Diversion Scheme”, simulated water consumption in the middle reaches of Heihe River under different inflow scenarios, and proposed the water resources regulation strategy to meet the needs of ecological water in the lower reaches of Heihe River.

## 2. Materials and Methods

### 2.1. Study Area

**Figure 1:**
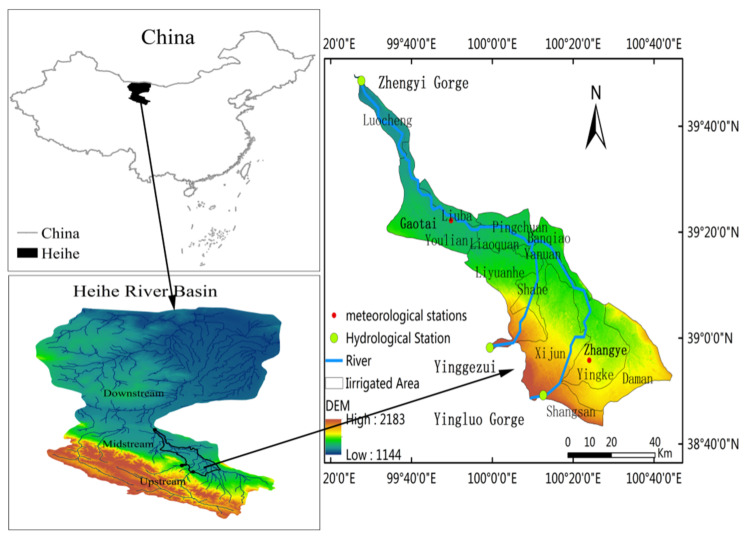
Study area.

The Heihe River is the second-largest inland water body in Northwest China, with the midstream located at 38.6° N–39.8° N, 99.5° E–100.8° E (Figure 1). Heihe is an important irrigated agricultural area. The average altitude of the Heihe River’s midstream is 1451 m, the annual average temperature is 6–8 °C, and the yearly precipitation is about 150 mm.

**Figure 2:**
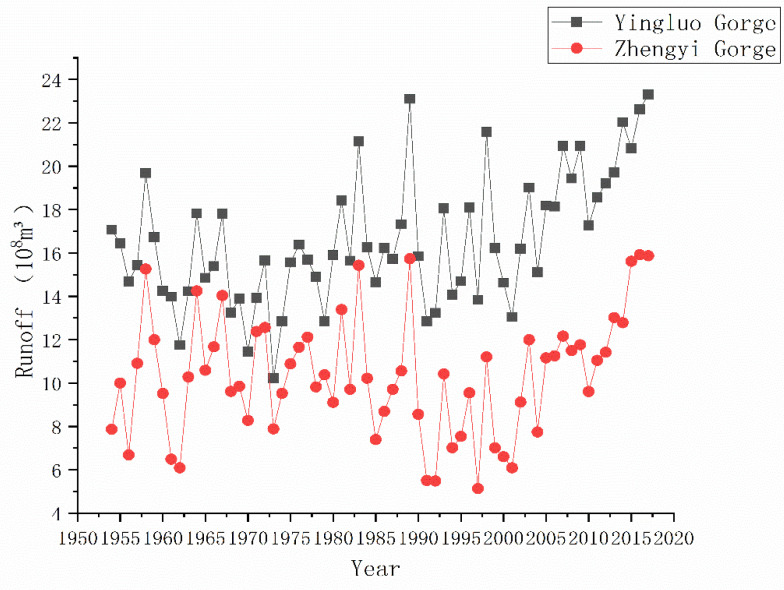
Interannual variation of runoff.

The Heihe River originates from the northern foothills of the Qilian Mountains. The upstream above Yingluo Gorge, the midstream between Yingluo Gorge and Zhengyi Gorge, and the downstream below Zhengyi Gorge. The “97 Water Diversion Scheme” strictly stipulates the inflow of water from Yingluo Gorge and the Zhengyi Gorge’s discharge under different inflow conditions. (Figure 2) “Three Red Lines” set strict rules on water consumption in significant districts and counties along the midstream of the Heihe River, limiting and complementing each other.

The midstream of the Heihe River includes three districts and counties (Zhangye City, Linze County, Gaotai County) and 13 irrigated areas (Shangsan, Daman, Yingke, Xijun, Shahe, Liyuanhe, Yanuan, Banqiao, Liaoquan, Pingchuan, Youlian, Liuba, Luocheng). Li Yuan River is a tributary of the Heihe River, the Liyuan River irrigated area irrigated by the Liyuan River, and the other 12 are irrigated by the Heihe River mainstream. Due to the development of the economy and agriculture in the midstream of the Heihe River, groundwater demand is also great, so groundwater is extracted by underground mechanical wells for agricultural irrigation.

**Figure 3:**
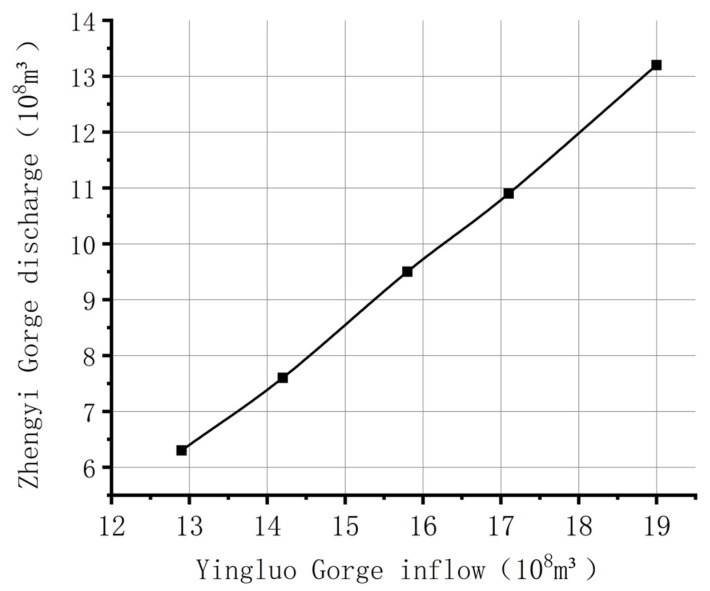
97 Water Diversion Scheme.

The Ministry of Water Resources approved the “Water Distribution Plan for the Mainstream of the Heihe River.” This plan allocates the water volume of the Heihe River in the wet and dry years (Figure 3). When the Yingluo Gorge has a 10% guaranteed rate of incoming water of 1.90 billion m^3^, the Zhengyi Gorge discharges 1.32 billion m^3^. When the Yingluo Gorge’s 25% guaranteed rate of incoming water is 1.71 billion m^3^, the water discharged from Zhengyi Gorge is 1.09 billion m^3^. When Yingluo Gorge’s 75% guaranteed rate of incoming water is 1.42 billion m^3^, the discharged water will be 760 million m^3^. When Yingluo Gorge’s 90% guaranteed rate of incoming water is 1.29 billion m^3^, the discharge volume of Zhengyi Gorge will be 630 million m^3^ [55].

**Figure 4:**
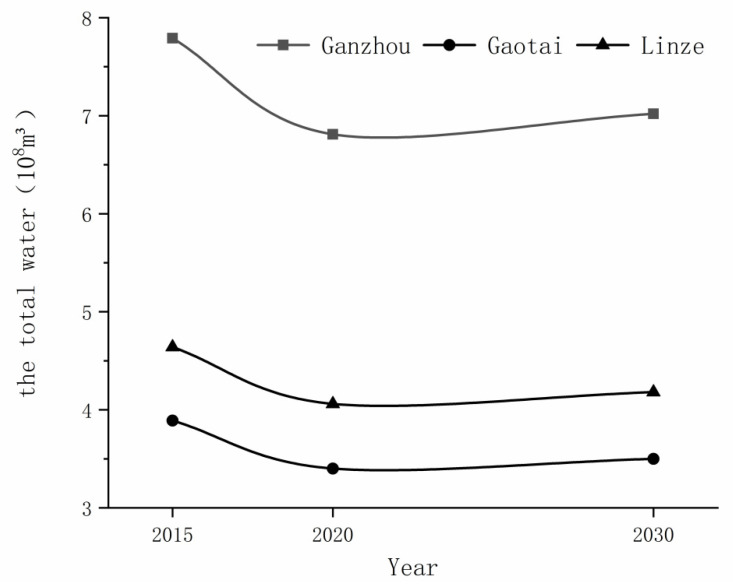
Total water consumption index.

In January 2013, the General Office of the State Council issued the most stringent “Three Red Lines” indicators for water resources management to all provinces, autonomous regions, and municipalities directly under the central government. In November, the general office of the Gansu Provincial People’s government issued water resources management control indicators for 2015, 2020, and 2030 for the Gansu Province prefecture-level administrative regions. According to the city water resources management target, the Heihe water diversion scheme, water resources allocation scheme, and water use practice for each county, Zhangye City issued its total water consumption targets in 2015, 2020, and 2030 (Figure 3). The total water consumption control indexes for Zhangye City were as follows: 2.3 billion m^3^ in 2015, 2.011 billion m^3^ in 2020, and 2.71 billion m^3^ in 2030. The total water consumption control indexes for Ganzhou District were 779 million m^3^, 681 million m^3^, and 702 million m^3^. The total water consumption control indexes for Linze County were 464 million m^3^, 406 million m^3^, and 418 million m^3^. The total water consumption control indexes for Gaotai County were 389 million m^3^, 340 million m^3^, and 350 million m^3^ [55,56] (Figure 4).

### 2.2. Data

The Cold and Arid Region Scientific Data Center provided annually observed runoff discharge (1956–2017) and Digital Elevation Model (DEM) (http://data.casnw.net accessed on 30 June 2020). We obtained meteorological data from six weather stations in the Heihe River watershed from 1956 to 2017 from the China National Meteorological Information Center (http://cdc.cma.gov.cn accessed on 8 July 2020). We obtained data for irrigated area, average flow rate, and the closing time of the river in the section from the Zhangye Water Conservancy Annals (http://www.zhangye.gov.cn accessed on 8 July 2020) and Gansu Statistical Yearbook (http://tjj.gansu.gov.cn accessed on 8 July 2020).

### 2.3. Method

#### 2.3.1. Water Resource Allocation Model in the Middle Reaches of the Heihe River

**Figure 5:**
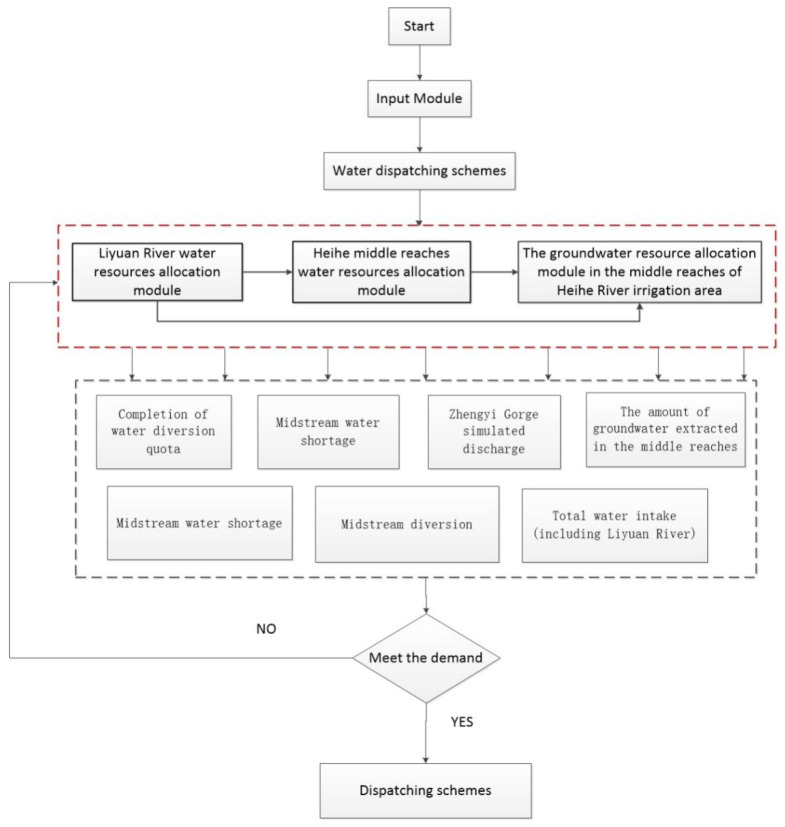
Framework of the water resource allocation model.

**Figure 6:**
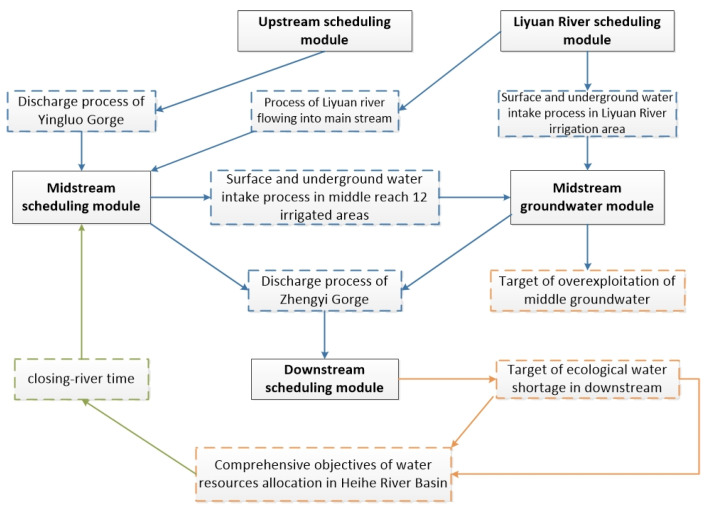
Operation process of water resource allocation model.

The utilization of water resources in the Heihe River Basin should comprehensively consider the ecological water demand of the irrigated areas of the midstream and the downstream. Therefore, we established two water resource allocation targets:

- Under the condition of satisfying the water demand guarantee rate of different types of users, reduce the over-exploitation of groundwater in the midstream.
- Strive to reduce the annual water shortage in the downstream ecological region of Langxin Mountain section (Figure 5 and Figure 6).

On this basis, we established two single objective functions:

(1) $$\min f_{M}=max\left(\frac{1}{n}\overset{n}{\underset{i=1}{\sum }}W_{g,i}-W_{g}^{P},0\right)$$

(2) $$\min f_{L}=max\left(W_{Ec}^{D}-\frac{1}{n}\overset{n}{\underset{i=1}{\sum }}W_{Ec,i}^{S},0\right)+\varphi_{1}\cdot max\left(W_{Ec}^{KD}-\frac{1}{n}\overset{n}{\underset{i=1}{\sum }}W_{Ec,i}^{KS},0\right)$$

where *f_M_* is the average over-recovery of middle groundwater for many years, 10^8^ m^3^; *f_L_* is the total amount of ecological water shortage considering the perennial average water shortage of Zhengyi Gorge and the perennial average water shortage of Langxin Mountain during the critical period of ecology, 10^8^ m^3^; *W_gj_* is the groundwater exploitation in the i year in the midstream, 10^8^ m^3^;${\;W}_{g}^{p}$ is the recoverable amount of groundwater in the midstream, 10^8^ m^3^;${\;W}_{Ec}^{D}$ is the downstream ecological water demand, 10^8^ m^3^;${\;W}_{Ec,i}^{S}$ provides water for the downstream ecology in I year, 10^8^ m^3^;${\;W}_{Ec}^{KD}$ is the water demand in the critical period of downstream ecology, 10^8^ m^3^;${\;W}_{Ec,i}^{KS}$ is the water supply in the critical period of the i-th downstream ecological period, 10^8^ m^3^; and *φ*_1_ is the coordination coefficient, which is an integer greater than 1, used to coordinate the relationship between the ecological annual water shortage and the water shortage in the critical period.

In Equation (2), the coordination coefficient (*φ*_1_) is an empirical parameter, which is determined after many trials and is combined with the Heihe water resources deployment.

The decision variable is the closing time of the channel. According to the water requirements during the critical period of ecological water demand, we selected the river channel’s closing time from April and August. The closed-mouth rate characterized the closing time, the closed-mouth rate is the ratio of the days of the closing time and the total days, with a value of 0–1.

#### 2.3.2. Model Parameter Calibration

The parameters of the Heihe River Basin water resource allocation model included the maximum allowable mining depth h_g,D_, the precipitation infiltration replenishment coefficient α, the canal water utilization coefficient η_q_, the field water utilization coefficient η_t_, the field infiltration replenishment coefficient β, the canal system leakage ratio coefficient m_r_, water supply μ, water conductivity T_g_, vertical permeability coefficient K_V_, phreatic flow slope width product d_g_, phreatic flow width-length ratio e_g_, phreatic evaporation empirical constant z, and phreatic evaporation limit buried depth $h_{g}^{\max}$. To improve the Heihe River Basin water resources allocation model’s operating efficiency, we divided these parameters into predetermined parameters and undetermined parameters. The predetermined parameter directly determined the value within a given range, including h_g,D_, α, η_q,_ η_t_, β, m_r_, K_V_, z, and $h_{g}^{\max}$.The undetermined parameters refer to the parameters whose values can be obtained through optimization calculation within a given range, including μ, T_g_, d_g_, and e_g_.

1. Maximum allowable mining depth h_g,D_

The burial depth of the groundwater in the irrigation area was the maximum allowable burial depth. If the irrigated area continued to mine groundwater, we considered it to be over-exploited. The maximum permissible mining depth was related to such factors as the irrigation area ecological health level, the natural characteristics of groundwater, and the method of groundwater extraction. Currently, no reasonable calculation method is available. In this project, we used the difference between the lowest groundwater level measured from 2005 to 2012 for each irrigation area in the midstream and used the surface elevation as the maximum allowable mining depth for each irrigation area.

2. Precipitation infiltration recharge coefficient α

The precipitation infiltration recharge coefficient was the ratio of precipitation to groundwater for a certain area during a specific period. The coefficient was affected by rainfall, lithology, groundwater depth, and moisture content of the vadose zone’s moisture content. We set the coefficient range at 0.1–0.2.

3. Canal water use coefficient η_q_ and field water use coefficient η_t_

The canal system water utilization coefficient was the ratio of the amount of water entering the field at the end of the canal system to the water taken from the canal head. The coefficient was related to factors such as the length of the canal system and the lining condition. The field water use coefficient was the ratio of water used by crops or forests and grasses to the amount of water entering the field. This coefficient was related to factors such as crop types and soil types. The canal water utilization coefficient and the field water utilization coefficient were the irrigation water utilization coefficient. In the current level year, the water use coefficient of the canal system in the midstream of the Heihe River was 0.52–0.61, the field water use coefficient was 0.9–0.92, and the irrigation water use coefficient was 0.47–0.56.

4. Field infiltration recharge coefficient β

The field infiltration recharge coefficient was the ratio of irrigation water quantity to net irrigation water quantity. This coefficient was related to such factors as groundwater depth, irrigation quota, and lithology. Considering the actual situation in the midstream of the Heihe River [56], we set the recharge coefficient range of field water seepage in the midstream of the Heihe River Basin as 0.28–0.46.

5. Supply coefficient of canal leakage m_r_

The supply coefficient of canal leakage was the canal system water’s ratio to the canal head water intake. This coefficient was related to climate, canal system lining, and lithology under the canal bed. The canal system leakage replenishment coefficient and the canal system water utilization coefficient were the same. This study, used the canal system leakage proportional coefficient instead of the canal system water leakage replenishment coefficient. The proportion coefficient of canal system leakage was the ratio canal system water supply to the amount of canal system water loss. We set the range of the canal system leakage ratio coefficient to be 0.4–0.6.

6. Water supply degree μ and conductivity coefficient T_g_

Water supply was the ratio of the maximum volume of water discharged from a saturated aquifer under gravity to the underground aquifer volume. According to the current situation for the Heihe River [56], the water supply degree range of the middle reaches of the Heihe River Basin was 0.1–0.35, and the water conductivity coefficient range was 300–6500 m^2^/d.

7. Vertical permeability coefficient K_V_

The vertical permeability coefficient was the speed at which surface water flows vertically through the vadose zone to reach the diving surface. The unit’s vertical permeability coefficient in the midstream of the Heihe River Basin was 0.65 m/d [57].

8. Slope width product of diving flow $d_{g}$ and width-to-length ratio of diving current e_g_

According to the surface contact boundary length of adjacent units in the middle reaches of the Heihe River Basin and the unit centroid distance [57], we set the phreatic flow slope product range from the outside to the irrigation area unit to be 0.5–30 m and set the phreatic flow width-to-length ratio range of the adjacent units to be 0.1–20.

9. Empirical constant of diving evaporation and limit depth $h_{g}^{\max}$

Taking into account the main crop types (wheat and corn) and the main soil types (loam) in the midstream of the Heihe River Basin [58,59], we set the empirical constant of diving evaporation at 2.6. According to the observation data of the second hydrological team of the Gansu Geological Bureau [60], we set the maximum buried depth of phreatic evaporation in the middle reaches of the Heihe River Basin to be 5 m.

Except for K_V_, z, and $h_{g}^{\max}$, which have the same fixed value in each irrigation area, other predetermined parameters of different irrigation areas are given in Table 1.

According to the groundwater module in the midstream of the Heihe River Basin water resources allocation model, we established an objective function using the historical three-year (2000, 2010, and 2012) average water withdrawal data, multiyear average precipitation, multiyear average water surface evaporation, and predetermined parameters of each irrigation area in the middle reaches, and optimized the calculation of pending parameters.

The objective function is as follows:

(3) $$\mathrm{minf}=\overset{m}{\underset{i=1}{\sum }}{\left(WI_{i}^{gq}-WO_{i}^{gq}\right)}^{2}+{\left(W_{r}^{gq}-2.7\right)}^{2}+{\left(W_{gq}^{r}-6.1\right)}^{2}+{\left(W_{jw}^{gq}-1.4\right)}^{2}$$

where *m* is the number of irrigation area units, including subirrigation area units, *m* = 23; ${WI}_{i}^{gq}$ and ${WO}_{i}^{gq}$ are the water input and output items of the i-th irrigation district unit, respectively, 10^8^ m^3^; $W_{r}^{gq}$ is the total amount of surface water used to replenish groundwater in the corresponding irrigation area in the Yinggao River section, 10^8^ m^3^; $W_{gq}^{r}$ is the total amount of surface water supplied by the unit groundwater in the irrigation area to the Gaoping reach and the Pingzheng reach, 10^8^ m^3^; ${\mathrm{and}\;W}_{jw}^{gq}$ is the total amount of groundwater in the corresponding irrigated area in the overseas groundwater recharge research area, 10^8^m^3^. We obtained the numerical values of undetermined parameters (μ, T_g_, d_g_, and e_g_) according to the optimization calculations given in Table 2, Table 3, Table 4 and Table 5:

## 3. Results

### 3.1. Rationality Analysis of Water Resources Allocation in the Middle Reaches of Heihe River

The model simulated the groundwater level of each irrigation area and the flow of each river section from 2005 to 2012. It was evident from the main section flow fitting and the groundwater level simulation that the results were better.

#### 3.1.1. The Middle Reaches of Heihe River Control Section Annual Runoff

**Figure 7:**
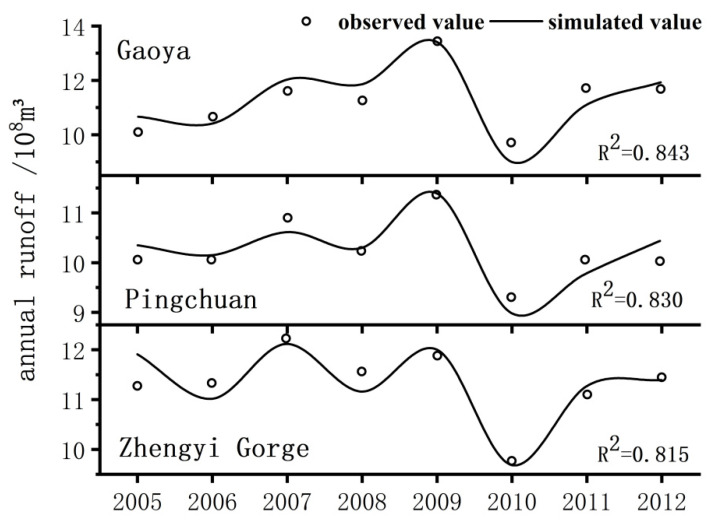
Annual runoff simulation of the controlled section of the middle reaches of Heihe River.

Figure 7 shows the annual runoff simulation process of the three midstream control sections of Gaoya, Pingchuan, and Zhengyi Gorge. The annual runoff simulation effect of the control section in the middle reaches of the Heihe River was good, and the degree of fit was between 0.81 and 0.85. Therefore, from the perspective of the annual runoff simulation effect of the control section in the middle reaches of the Heihe River, the groundwater balance model and optimal parameter values were reasonable.

#### 3.1.2. Annual Average Groundwater Level in the Middle Reaches of the Heihe River Irrigation Area

**Figure 8:**
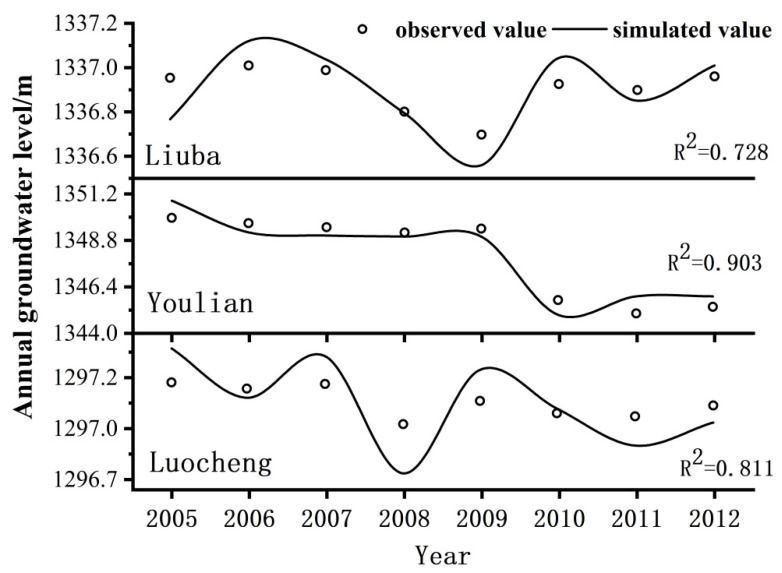
Simulation of the groundwater level in the middle reaches of the Heihe River from Pingchuan to Zhengyi Gorge.

**Figure 9:**
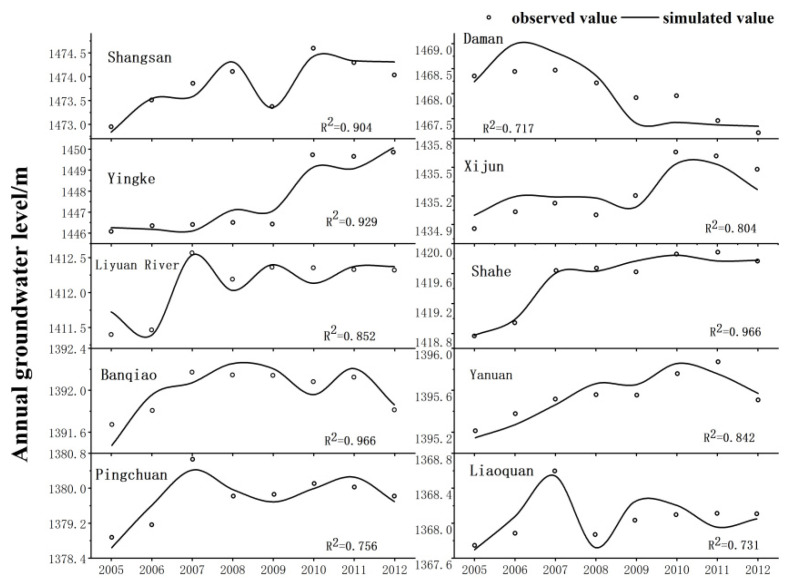
Simulation of groundwater level in the middle reaches of the Heihe River from Yingluo Gorge to Pingchuan River.

Figure 8 and Figure 9 show the groundwater simulation results of 13 irrigation districts in the middle reaches of the Heihe River (from Yingluo Gorge to Zhengyi Gorge). The fitting degree of the annual average groundwater level of the four irrigation districts of Shangsan, Yingke, Shahe, and Youlian was greater than 0.9. The fitting degree of the annual average groundwater level of the four irrigation districts of Xijun, Liyuanhe, Yanuan, and Luocheng was greater than 0.8. The fitting degrees of the average annual groundwater levels of the other five irrigation districts, including Manchu and Banqiao, were all above 0.7. Therefore, from the perspective of the average annual groundwater level in the middle reaches of the irrigation area, the groundwater balance model and parameter optimization values in the middle reaches of the Heihe River were reasonable.

### 3.2. Water Resources Allocation in the Middle Reaches of the Heihe River Without Considering the Total Water Consumption Index Control

In 2017, the midstream of Heihe needed 1.394 billion m^3^ of water. We used the water allocation to simulate the completion of a long series of annual water diversion schemes under current water demand conditions. Table 6 shows the simulation results of a long series of years (1956–2017), without considering the total water consumption control under current water demand conditions. According to the table, during years when water was abundant, the simulated discharge of Zhengyi Gorge was less than the discharge index. In wet years, 164 million m^3^ of downstream water was owed annually. In more abundant years, 126 million m^3^ water was owed to downstream water. In a dry year and normal flow year, the discharge target of the Zhengyi Gorge could be met. The average annual groundwater extraction volume was 598 million m^3^, which was greater than the average annual extraction volume of 480 million m^3^ in the middle reaches of the Heihe River [28]. The average level of over-extractions was 23% over-extraction.

By comparing the Zhengyi Gorge’s drainage index with the Zhengyi Gorge simulated discharge, under different incoming water conditions, the Zhengyi Gorge simulated discharge is less than the drainage index, and Midstream water consumption is greater than the water consumption index. Under such circumstances, the ecological water in the downstream will be insufficient, thus affecting the ecological environment in the downstream. Total water withdrawal (including Liyuan River) in the midstream is also greater than the Midstream water consumption index. With the increase of water consumption in the study area, the extraction of water from Heihe River will increase, and the discharge from Zhengyi Gorge will become smaller, which is more detrimental to the ecological environment of the downstream of the Heihe River.

In each inflow year, the total water intake was greater than the total water withdrawal index (1.63 billion m^3^), and the total water consumption also exceeded the midstream water consumption index. Therefore, under current water demand conditions, if the total water consumption was not taken into account, through the reasonable allocation of water resources, the water intake and consumption would exceed the indexes of in the middle reaches of “97 Water Diversion Scheme” in the Heihe River. As a result, the ecological water demand of the lower reaches of the Heihe river decreases, which affects the ecological development of the lower reaches of the Heihe River.

### 3.3. Water Resources Allocation in the Middle Reaches of Heihe River Considering Control of Total Water Consumption Index

Table 7 provides the simulation results for the long series of years (1956–2017) considering total water consumption control. According to the table, in different inflow years, the simulated discharge of Zhengyi Gorge was basically equivalent to the discharge index, and the simulated discharge could complete the drainage index of Zhengyi Gorge. In different inflow years, the total water intake index was lower than the total water intake index of the three counties (1.63 billion m^3^), and total water consumption and total water consumption indicators were basically equivalent. Therefore, considering the water consumption, under current water requirements, through water allocation, the water intake and consumption in the middle reaches of Heihe River could be controlled within the “Three Red Lines” for water intake and consumption indicators.

By comparing the Zhengyi Gorge’s drainage index with the Zhengyi Gorge simulated discharge, under different incoming water conditions, the Zhengyi Gorge simulated discharge is less than the drainage index. However difference between the results is not significant. Total water withdrawal (including Liyuan River) in the midstream is also less than the Midstream water consumption index. By the conditions, the discharge volume of Zhengyi Gorge can be satisfied to meet the downstream ecological water.

Under these circumstances, the water shortage in the middle reaches was significantly greater than without the considering water consumption index. In years when the incoming water was 10%, 25%, 50%, 75%, and 90%, the water shortage was 1.64 and 1.61, 1.55, 1.58, and 1.56 million m^3^.

In the case of considering water consumption, the downstream ecological water demand can be satisfied through rational allocation of water resources, so as to achieve the purpose of water resources allocation, but the water shortage in the middle reaches was significantly greater than without considering the water consumption index. So some measures should be taken.

## 4. Discussion

According to the results of water resources allocation, we obtained different results under different inflow conditions. If the total water consumption limit was not taken into account, the water consumption in the middle reaches of the Heihe River exceeded the water consumption target. If the total water consumption limit was not taken into account, the water shortage in the middle reaches significantly increased. We identified two solutions: (1) Returning farmland to 1200 Km^2^; (2) use advanced technologies to save water and improve the utilization efficiency of water resources, to increase the utilization coefficient of irrigation water to 0.68. Increasing water-saving measures can reduce water consumption and improve water efficiency.

### 4.1. Returning Agricultural Land to 1200 Km^2^

The increase in the cultivated land area has been the main factor driving water demand. Reducing midstream water demand can achieve the water diversion target in wet years. The cultivated land area in the middle reaches should be 1200 km^2^, and the corresponding water demand is 1.023 billion m^3^ (Heihe Recent Governance Plan) [28]. We simulated the completion of a long series of annual water separation indexes under the water demand scheme. Table 8 shows that the water diversion index can be completed in different inflow years, and the average annual groundwater exploitation was 528 million m^3^, with 10% over-extraction. The water consumption in the middle reaches of Heihe could be controlled within the “97 Water Diversion Scheme”.

### 4.2. On the Premise of Maintaining the Farmland Area of 1200 Km^2^, Water-Saving Measures Were Implemented to Increase the Utilization Coefficient of Irrigation Water to 0.68

Strengthening water saving in the middle reaches can alleviate the water contradiction of water use in the middle reaches. In the middle reaches of Heihe River, the water demand was 1.15 billion m^3^ under water-saving conditions. In addition, the reclaimed farmland in the middle reaches of the Heihe River to 1200 Km^2^ and the corresponding water demand was 1.046 billion m^3^ [28]. We simulated the completion of a long series of annual water separation indexes under the water demand scheme. Table 9 shows that the water diversion index could be completed in different inflow years, and the average annual groundwater exploitation amount was 549 million m^3^, with 14% over-extraction. The water consumption in the middle reaches of the Heihe River could be controlled within the “97 Water Diversion Scheme”.

According to our results, if water consumption and water use were controlled, farmland was returned, and water-saving measures were applied, the indicators of the middle reaches of the Heihe River could be met. The over-exploitation of groundwater could also be categorized as mild over-exploitation. The water shortage was not significant, and water resources allocation in the middle reaches of the Heihe River was well satisfied. So, the application of returning farmland and water-saving measures is an important measure for water resources allocation in the Heihe River, and controlling the scale of farmland is the most important.

## 5. Conclusions

On the basis of our simulation, we made the following conclusions.

- The “97 Water Diversion Plan” and the “Three Red Lines” control the consumption and water use, respectively, in the middle reaches of the Heihe River. With improvements in water use level, and without exceeding the water use targets, water consumption still increased. This affected the ecological water of the lower reaches of the Heihe River. We constructed a water resource allocation model to satisfy the “97 Water Diversion Plan” and solve the shortage of ecological water of the lower reaches.
- Without considering the total water consumption index, even though the reasonable allocation of water resources, water intake, and consumption in the middle reaches of the Heihe River exceeded the water intake and consumption indicators. Considering water consumption in the middle reaches, and with the allocation of water resources, the water intake and consumption could be controlled within the indicators.
- After the rational allocation of water resources, it is necessary to control the farmland area to 1200 Km^2^ and implement water-saving measures, especially the control of farmland area is of great significance for the Heihe river resources.

## Figures and Tables

**Table 1:** Specific numerical values of some predetermined parameters of each irrigation area in the midstream.

Irrigated Area	$h_{g,D}$ (m)	*α*	*η*_q_^1^	*η*_t_^1^	*β*	*m_r_*
Shangsan	154.6	0.10	0.52	0.92	0.29	0.40
Daman	20.1	0.12	0.58	0.92	0.40	0.44
Yingke	16.3	0.13	0.58	0.92	0.41	0.47
Xijun	10.5	0.16	0.58	0.92	0.44	0.52
Liyuan river	2.7	0.19	0.61	0.91	0.46	0.58
Shahe	35.1	0.10	0.60	0.91	0.34	0.40
Banqiao	11.8	0.15	0.52	0.91	0.43	0.51
Yanuan	4.1	0.18	0.54	0.91	0.46	0.57
Pingchuan	5.2	0.18	0.58	0.91	0.46	0.56
Liaoquan	4.4	0.18	0.57	0.91	0.46	0.56
Liuba	3.6	0.19	0.60	0.90	0.46	0.57
Youlian	11.1	0.16	0.58	0.90	0.43	0.51
Luocheng	3.2	0.19	0.60	0.91	0.46	0.57

^1^ η_q_ and η_t_ are the current level year data. The η_q_ and η_t_ of the short-term and long-term level years are enlarged in proportion to the average irrigation water utilization coefficient; the predetermined parameters of the sub-irrigation area units with the same name and different numbers are the same.

**Table 2:** Optimal value of degree of unit water supply in the middle irrigation area.

Irrigation Unit	*μ*	Irrigation Unit	*μ*	Irrigation Unit	*μ*	Irrigation Unit	*μ*
Shangsan	0.31	Xijun1	0.11	Yanuan1	0.10	Liuba	0.30
Daman	0.12	Xijun2	0.13	Yanuan2	0.19	Youlian1	0.12
Yingke1	0.35	Liyuan river 1	0.32	Pingchuan1	0.30	Youlian2	0.13
Yingke4	0.21	Liyuan river 2	0.10	Pingchuan2	0.30	Luocheng1	0.23
Yingke2	0.15	Shahe	0.28	Liaoquan1	0.23	Luocheng2	0.32
Yingke3	0.12	Banqiao	0.26	Liaoquan2	0.12		

**Table 3:** Optimal values of slope width product and water conductance of submersible flow from outside to an irrigated area.

Overseas— Irrigated Unit	*d_g_* (m)	*T*_g_ (m^2^/d)	Overseas— Irrigated Unit	*d*_g_ (m)	*T*_g_ (m^2^/d)
Overseas—Shangsan	5.7	3291	Overseas—Pingchuan 1	6.9	3551
Overseas—Daman	16.7	855	Overseas—Pingchuan 2	14.6	5002
Overseas—Yingke 4	4.9	2044	Overseas—Liuba	3.3	4997
Overseas—Xijun 1	1.3	828	Overseas— Youlian 1	11.0	2741
Overseas—Liyuan river 1	26.4	3370	Overseas— Youlian 2	3.6	2829
Overseas—Liyuan river 2	0.5	1036	Overseas—Luocheng 1	4.7	4084
Overseas—Banqiao	10.0	3064	Overseas—Luocheng 2	10.5	5252

**Table 4:** The optimal value of the width-to-length ratio and the coefficient of water conductance between the irrigated area and the channel.

Irrigated Unit-Streamway	*e*_g_	*T*_g_ (m^2^/d)	Irrigated Unit-Streamway	*e*_g_	*T*_g_ (m^2^/d)
Shangsan-streamway	2.0	3088	Pingchuan 1-streamway	0.1	5181
Danman-streamway	14.0	702	Pingchuan 2-streamway	16.2	3441
Yingke 1-streamway	0.8	3536	Liaoquan 1-streamway	5.7	4195
Yingke 4-streamway	1.1	2114	Liaoquan 2-streamway	6.9	882
Yingke 2-streamway	14.0	1066	Liuba-streamway	1.6	3437
Yingke 3-streamway	4.2	2681	Youlian 1-streamway	17.4	932
Xijun 1-streamway	16.9	675	Youlian 2-streamway	0.4	1029
Banqiao-streamway	21.3	4640	Luocheng 1-streamway	17.4	2423
Yanuan 2-streamway	16.7	3688	Luocheng 2-streamway	22.2	3719

**Table 5:** The optimal value of the width-to-length ratio of the diving flow and the water conductivity coefficient of the adjacent irrigated area unit.

Adjacent Irrigated Area Unit	*e*_g_	*T*_g_ (m^2^/d)	Adjacent Irrigated Area Unit	*e*_g_	*T*_g_ (m^2^/d)
Shangsan–Daman	15.0	3151	Liyuan River 2–Yanuan 1	17.8	307
Damna–Yingke 1	4.8	3608	Liyuan River 2–Liaoquan 2	13.4	529
Daman–Yingke 4	7.8	1904	Liyuan River 2–Youlian 1	0.7	574
Yingke 1–Yingke 4	0.2	4796	Shahe–Yanuan2	0.2	3675
Yingke 2–Yingke 3	8.7	1150	Yannuan 1–Liaoquan 1	5.0	1933
Yingke 4-Banqiao	2.7	3731	Yanuan 1–Yanuan 2	14.3	1477
Yingke 3–Yanuan 2	15.6	1747	Banqiao–Pingchuan 1	4.2	4857
Xijun 1–Xijun 2	9.4	900	Pingchuan 1–Pingchuan 2	7.2	5350
Xijun 1–Liyuan River 1	0.1	3203	Liaoquan 1–Liaoquan 2	8.1	2155
Xijun 2–Yingke 2	10.2	1299	Lianquan 2–Youlian 1	13.8	789
Xijun 2–Yingke 3	8.6	989	Pingchuan 2–Liuba	0.5	5352
Xijun 2–Shahe	15.5	2918	Liuba–Youlian2	1.8	3179
Xijun 2–Yanuan 2	3.5	1896	Youlian 1–Luocheng 2	0.4	3345
Liyuan River 1–Shahe	0.6	5221	Youlian 2–Luocheng 1	4.6	2266
Liyuan River 2–Shahe	1.5	2505			

**Table 6:** Under the condition of current water demand, the allocation results of midstream water resources controlled by water consumption index were not considered, 10^8^ m^3^.

Water-Coming Year	Inflow from Ying luo Gorge	Zhengyi Gorge Drainage Index	Zhengyi Gorge Simulated Discharge	Completion of Water Diversion Quota	The Amount of Groundwater Extracted in the Middle Reaches	Midstream Water Shortage	Midstream Diversion	Total Water Withdrawal (Including Liyuan River)	Midstream Water Consumption	Total Water Withdrawal Index	Midstream Water Consumption Index
10%	20.27	14.73	13.09	−1.64	6.81	0.52	9.22	17.29	6.48	16.3	5.53
25%	17.69	11.63	10.37	−1.26	6.28	0.28	10.10	17.64	6.65	16.3	6.06
50%	15.63	9.29	9.22	−0.07	6.03	0.12	10.56	17.85	6.41	16.3	6.34
75%	14.56	8.10	7.8	−0.30	5.65	0.24	10.77	17.68	6.76	16.3	6.46
90%	12.87	6.28	6.32	0.04	5.48	0.11	10.98	17.72	6.55	16.3	6.59

**Table 7:** The results of mid-stream water resources allocation under the condition of current water demand are considered, 10^8^m^3^.

Water-Coming Year	Inflow from Ying luo Gorge	Zhengyi Gorge Drainage Index	Zhengyi Gorge Simulated Discharge	Completion of Water Diversion Quota	The Amount of Groundwater Extracted in the Middle Reaches	Midstream Water Shortage	Midstream Diversion	Total Water Withdrawal (including Liyuan River)	Midstream Water Consumption	Total Water Withdrawal Index	Midstream Water Consumptin Index
10%	20.27	14.73	14.70	−0.03	6.81	1.64	8.09	16.17	5.57	16.3	5.53
25%	17.69	11.63	11.37	−0.16	6.28	1.68	8.69	16.24	6.22	16.3	6.06
50%	15.63	9.29	9.22	−0.07	6.03	1.55	9.12	16.42	6.41	16.3	6.34
75%	14.56	8.10	8.00	−0.1	5.65	1.58	9.42	16.34	6.56	16.3	6.46
90%	12.87	6.28	6.29	0.01	5.48	1.56	9.52	16.27	6.58	16.3	6.59

**Table 8:** The result of the allocation of water resources in the middle reaches was 120 km^2^ of farmland, 10^8^ m^3.^

Water-Coming Year	Inflow from Ying luo Gorge	Zhengyi Gorge Drainage Index	Zhengyi Gorge Simulated Discharge	Completion of Water Diversion Quota	The Amount of Groundwater Extracted in the Middle Reaches	Midstream Water Shortage	Midstream Diversion	Total Water Withdrawal (including Liyuan River)	Midstream Water Consumptin	Total Water Withdrawal Index	Midstream Water Consumptin Index
10%	20.23	14.41	14.21	−0.2	6.29	0.25	8.57	16.05	16.3	6.02	5.82
25%	18.08	12.31	11.96	−0.35	5.7	0.38	9.23	16.19	16.3	6.12	5.77
50%	15.91	9.6	9.72	0.12	5.46	0.43	9.34	16.13	16.3	6.19	6.31
75%	14.08	7.52	7.55	0.03	4.99	0.47	9.97	16.35	16.3	6.53	6.56
90%	12.02	5.42	5.57	0.15	4.97	0.53	10.31	16.74	16.3	6.45	6.6
average	16.16	9.93	9.88	−0.05	5.28	0.38	10.96	16.24	16.3	6.28	6.23

**Table 9:** The results of mid-stream water resources allocation under the condition of strong water-saving, 10^8^ m^3.^

Water-Coming Year	Inflow from Ying luo Gorge	Zhengyi Gorge Drainage Index	Zhengyi Gorge Simulated Discharge	Completion of water Diversion Quota	The Amount of Groundwater Extracted in the Middle Reaches	Midstream Water Shortage	Midstream Diversion	Total Water Withdrawal (including Liyuan River)	Midstream Water Consumptin	Total Water Withdrawal Index	Midstream Water Consumptin Index
10%	20.23	14.41	13.91	−0.5	6.43	0.26	8.24	15.86	16.3	6.32	6.6
25%	18.08	12.31	11.71	−0.6	5.64	0.31	9.12	16.02	16.3	6.37	6.56
50%	15.91	9.6	9.42	−0.18	5.43	0.27	9.53	16.29	16.3	6.49	6.31
75%	14.08	7.52	7.49	−0.03	5.31	0.3	9.77	16.47	16.3	6.59	5.77
90%	12.02	5.42	5.55	0.13	5.18	0.4	9.92	16.56	16.3	6.47	5.82
average	16.16	9.93	9.91	−0.02	5.49	0.3	10.77	16.26	16.3	6.25	6.23

## Data Availability

The data presented in this study are available on request from the corresponding author. The data are not publicly available due to privacy.
